# Chemical activation of kaolin-based clay bricks as a sustainable route to enhanced mechanical and thermophysical properties

**DOI:** 10.1038/s41598-026-35471-z

**Published:** 2026-02-02

**Authors:** Wafaa Soliman, M. Abdelhamid Shahat

**Affiliations:** 1 Geology Department Faculty of Science , Sohag University , Sohag, Egypt; 2 PV Unit, Solar and Space Research Department, National Research Institute of Astronomy and Geophysics (NRIAG), Helwan, Cairo, Egypt

**Keywords:** Fired clay-based composite bricks, Kaolin doping, Acid treatment, Thermophysical qualities, Thermal insulation, Sustainable development, Climate sciences, Environmental sciences, Materials science

## Abstract

This work examines the usage of various acid activators, including hydrochloric acid (HCl), sulphuric acid (H_2_SO_4_), phosphoric acid (H_3_PO_4_), and a combination of them, to improve the thermophysical properties of fired clay-based composite bricks modified-kaolin. To enhance these materials’ insulating capabilities, the main focus is on reducing their diffusivity, specific heat capacity, and thermal conductivity. Whereas, fibrous clays’ surface and catalytic properties were enhanced chemically via the addition of acid-activated kaolinite clay. The mechanical, thermophysical, morphological, shrinkage, density, porosity, microstructure, and shrinkage of each clay–kaolin(acid) composite were thoroughly examined, and the thermal conductivity performance was maximized. All of the peaks’ intensities in the XRD pattern increased in comparison to the untreated peak when varying acid types were added to the kaolin matrix. In the meantime, the addition of these activators caused the compositions’ apparent porosity (i.e., 29.15–29.47%) and compressive strength (i.e., 11.59–12.33 kg/cm^2^). The results demonstrate that treatment with these acids reduces thermal conductivity (i.e., 0.46–0.44 W/mk), and diffusivity, attributed to the increased porosity and altered microstructure of the bricks. Moreover, combining all three acids (H_2_SO_4_/HCl/H_3_PO_4_) resulted in the most significant improvements, yielding a composite with superior insulation capabilities. The aforementioned observations are attributed to the formation of two key mineral phases within the fired bricks: mullite and diopside. Mullite strengthens the bonding within the aluminosilicate framework, thereby enhancing the ceramic network and promoting a denser and more mechanically stable microstructure without causing a significant increase in porosity. Meanwhile, diopside also contributes to strength development and is widely recognized for its role in insulation ceramics due to its excellent thermal stability and chemical corrosion resistance.

## Introduction

A modest amount of water may transform the earthy, fine-grained natural substance known as clay into a flexible form. During the chemical and mechanical disintegration of silicate rocks, such as granite, in hot and humid conditions, a complex combination known as aluminosilicate containing bound water molecules forms^1^. Natural sediments, rocks with sedimentary compositions, hydrothermal deposits, and solids are the most common locations for clay^2^. In this regard, the clay brick industry has grown significantly over time as a result of emerging countries’ growing requirements for infrastructure as well as housing. As a result, the speedy industrialization of these countries has generated a large amount of rubbish that is harmful to the environment. To address this issue, several methods were employed to produce environmentally friendly bricks, one of which was a cementation and activation strategy that incorporated kiln fire. This approach replaces the traditional burnt clay bricks with clay-based bricks that were partly activated by acid. This gave rise to acid-activated kaolin bricks, which were mixed, shaped, and cured at both a regular or elevated temperature. Indeed, kaolin is a mineral-based clay that is typically white, earthy to dull, and pliable to the touch. According to its low specific surface area and relatively large particle size, kaolin shows reduced cohesion, swelling, and flexibility. Al_2_O_3_·2SiO_2_·2H_2_O is the chemical formula of this industrial rock. Kaolin deposits are used industrially for fired clay bricks, coating, and filling paper, as well as for paint, plastic, and ceramic raw materials for porcelain and tableware^3^. However, kaolin encounters to be modified in order to become an even more potent catalytic support. Chemical activation is one of the methods used to modify the properties of kaolin^4,5^.

According to Panda and et al.^6^, clay samples were leached using acid treatment, a type of chemical activation. This has resulted in the decomposition of clay systems, the removal of volatile materials and mineral contaminants, an increase in surface area, and the dissolution of the outer layers. It was successfully demonstrated that the dissolution or reordering of structural ions may outcome in modifications in the crystalline makeup of aluminosilicate minerals^7^. All of these processes have impacted the substance’s chemical makeup and arrangement. These changes also improve the clay’s catalytic properties by increasing the number of acid centers, contingent on the degree of treatment^8^. Alumina, silica, and fillers are the main components of clay, and they are all activated by acid-activators, particularly hydrochloric acid (HCl), phosphoric acid (H_3_PO_4_), and sulfuric acid (H_2_SO_4_)^9^. More details, variable cations in kaolin undergo replacement by H^+^ ions upon processing with HCl acid, and Al^3+^ progressively diffuses from both of these two sites. By increasing the material’s pore width, PH level, and porous zone, this boosts its mineralogical makeup and makes it a valuable raw material for the production of aesthetically beneficial ceramics and refractory bricks^10^. Given its unique properties, kaolin raises porosity and surface area by being more soluble in sulfuric acid^6^. Whereas, processing eliminated Al^3+^ ions, boosting the component’s porosity; nevertheless, the surface area and pore volume of the original kaolinite might vary substantially depending on processing both in time and temperature^11^. Similarly, phosphoric acid boosts its durability and purity rating by eliminating iron from kaolin clay. Whenever relative with alternative acids, the advantages of using phosphoric acid involve its use as a leaching agent and its ability to be diluted quickly to reach an elevated purity value^12^. Meanwhile, minerals enhanced the durability and functionality in clay by removing alumina and other metallic oxides^8^^13–15^,–^16^. Several research in the literature have used different acids or alkalis to activate pure kaolin, making it acceptable for a wide range of uses^17–21^. Belver and et al.^18^, modified pure kaolin via the inclusion of acids (i.e., HCL) and alkalis. Acid-activated kaolin eliminated over 90% of the octahedral Al^3+^ cations, yielding high surface area amorphous silica substance with tiny pores, whereas alkali activation enabled the development of massive zeolite structures^18^. Behnamfard et al.^19^, boosted the adsorptive features of kaolin by acid processing (i.e., sulfuric acid), thereby having a positive influence on its capacity to adsorb cyanide and purify aqueous liquids^19^. Cheng et al.^20^, described a phosphoric acid-kaolin treatment that significantly increased the kaolin’s ability to absorb alkaline minerals while also reducing ultrafine particles emitted via cornstalk pellet combustion^20^. Zhang and et al.^22^, developed porous bricks with a high compressive strength and dense microstructure by triggering kaolinite with phosphoric acid, while Abd El-Moghny and et al.^17^ activated kaolin with sodium hydroxide, an alkali, and utilized as a green building material in the formulation of geopolymers.

Despite the aforementioned characteristics of acid activators, there are still little research studies on their impact on the thermal characteristics of kaolinite-based burned clay bricks. Based on our knowledge, unique clay composites including, Clay@Kaolin@H_2_SO_4_, Clay@Kaolin@HCl, Clay@Kaolin@H_3_PO_4_, Clay@Kaolin@H_2_SO_4_/HC/H_3_PO_4_ were produced for the first time in this work to boost the thermophysical behaviour of bricks. These activators were specifically designed to promote the formation of key mineral phases within the bricks—namely mullite and diopside—which develop unique interactions with the clay matrix, enabling better regulation of moisture within the composite structure and consequently enhancing thermal performance. Mullite strengthens the bonding between the structural components, leading to a more homogeneous and mechanically robust framework^23,24^. Meanwhile, diopside promotes product strength^25^, serves in the creation of thermal insulators^26^, and plays an essential role in chemical corrosion resistance^27^. At this point, toxicity remains below the permissible level, and the bricks are extremely sensitive to manufacturing processes. The outcomes of this study target practical applications in sustainable construction, particularly in thermally challenged environments such as Upper Egypt and similar climates.

## Materials and methods

### Geologic setting

**Fig. 1:**
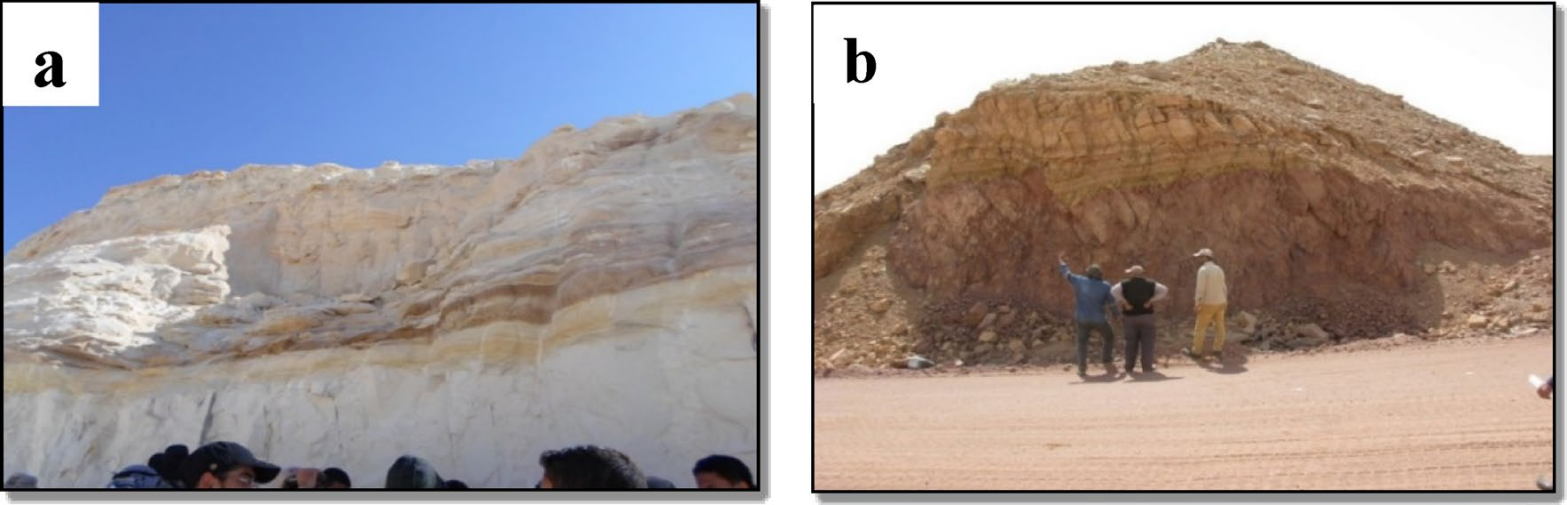
(**a**) Geological sample of Kalabsha kaolin collected from Wadi Kalabsha, Aswan Governorate. (**b**) The Quseir Formation exposed at the entrance of Abu Tartur Mine, Kharga Oasis, Western Desert of Egypt, showing the geological origin of the studied clay raw materials.

The extracted kaolin was derived from the Wadi Kalabsha area of the Aswan Governorate, which is precisely located at coordinates 24.088938^°^N latitude and 32.899830^°^E longitude (See Fig. 1a)^28,29^. The studied area is flat and covered in weathering products, with outcrops of kaolin beds. The study area’s eastern and southern regions contain a few small, elevated ridges and hills. Lower sandstone, Kalabsha kaolin, and upper sandstone are the three members of the stratigraphic section at Wadi Kalabsha that split from base to top. The kaolin under study is thought to be a clastic deposit that was created by the rocks’ aluminous parents weathering intensely, being transported, and then being deposited in an aqueous body. Whereas Kaolin is separated into four categories based on texture^30^. The clay utilized in brick development is Quseir formation, sourced from Kharga Oasis and Abu Tartur mine (25^°^26′18″N 30^°^33′30″E) (See Fig. 1b). The Quseir formation consists of multicoloured claystone with sandstone pockets. The Quseir Formation can be found along the foot scarp in the Abu Tartur area. It is distinguished by multicoloured claystone with sandstone pockets. It appears near Abu Tartur Mine and ranges in colour from grey, red, purple, and green. Table 1. summarizes the mineralogy composition of the clay raw material.

**Table 1.** Mineralogy of the clay raw material.

Bulk Mineralogy	Phyllosilicates (20%)	Quartz (7%)	Feldspars (70%)	graphite (3%)
Clay Fraction (˂ 2 µ)	smectite/illite mixed layers (S/I) (91%)		Kaolinite (9%)	

### Kaolin activation

The Kaolin activation procedure involved adding 10 ml of each acid inducing, sulphuric (H_2_SO_4_; 98% concentrated), hydrochloric (HCl; 37%), phosphoric (H_3_PO_4_; 85%), and mixture of the three acids (i.e., H_2_SO_4_/HCl/H_3_PO_4_) to the Kaolin suspension and leaving it on a magnetic stirrer for 4 h. Finally, the suspension was allowed to settle overnight before being filtered, dried, and ground into a fine powder using a mortar pastel.

### Design of the clay bricks

**Fig. 2:**
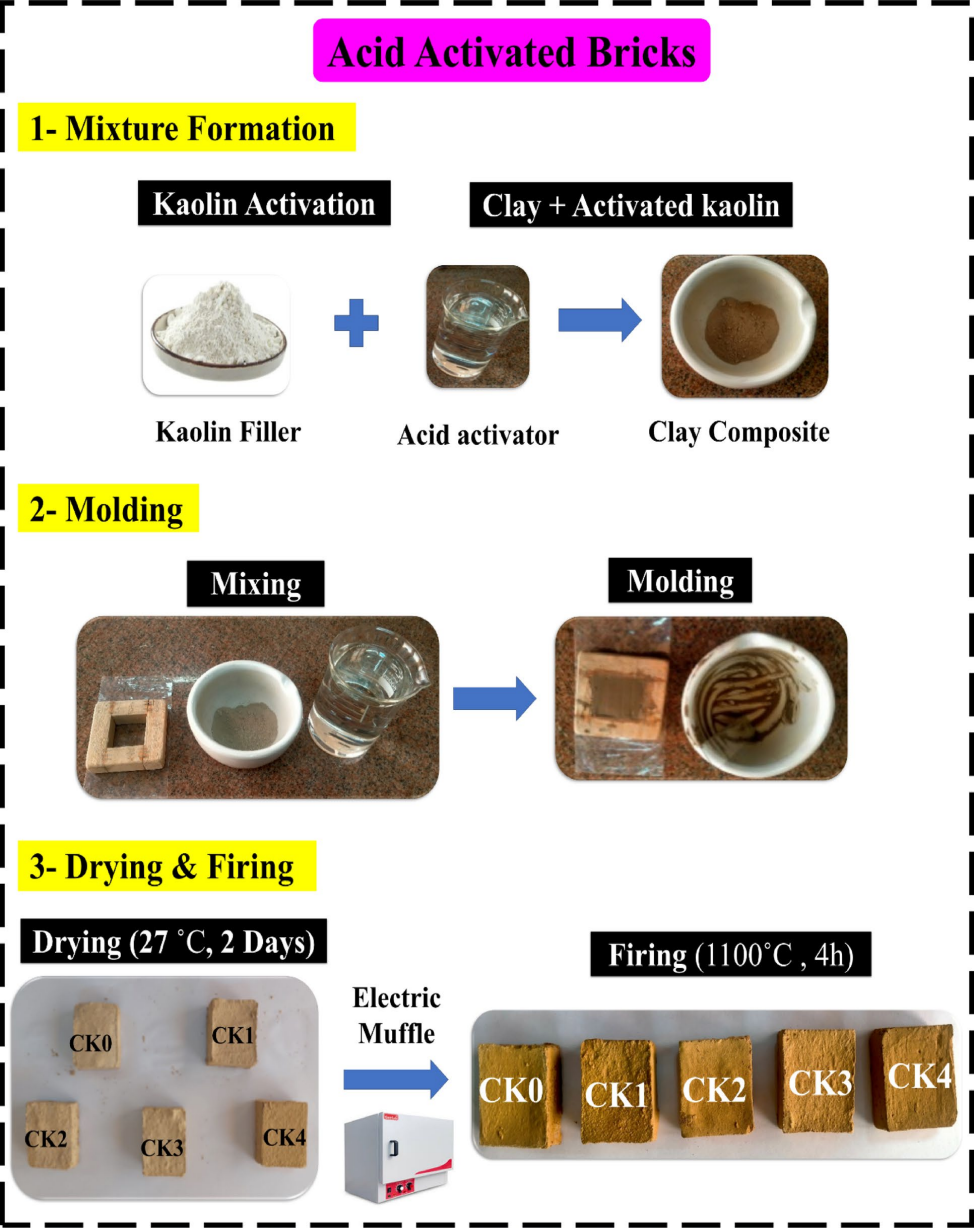
Schematic representation of the step-by-step process used for constructing and preparing the brick molds prior to shaping and firing.

Figure 2 highlights the process for producing clay bricks using various kinds of acid-modified kaolin, where each brick is composed of 40 g of tempering water, 49 g of clay, and 1 g of acid-activated kaolin; the corresponding mixing proportions of acid, water, and kaolin/clay for each sample are detailed in Table 2. First, 50 g of pure clay were used to make one brick; second, 49 g of clay and 1 g of kaolin/H_2_SO_4_; and third, 49 g of clay and 1 g of kaolin/HCl. The fourth brick is made up of 1 g of kaolin/H_3_PO_4_ and 49 g of clay. The fifth brick is made of one gramme of kaolin and 49 g of clay mixed with three acids. The untreated specimen is referred to as CK0, and those that were treated are referred to as CK_(H2SO4)_, CK_(HCl)_, CK_(H3PO4)_, and CK_(mix)_. Table 2 provided a detailed description of modified clay bricks rendered with acid-modified kaolin. In wooden molds (4 cm * 4 cm * 2 cm), the bricks were manually shaped. These clay bricks molded well and dried without developing any cracks. The bricks were allowed to air dry for two days before being fired for 4 h at 1100˚C in an electric muffle. Since quartz and oxides melt inside the fired specimens, firing at 1100˚C results in a higher vitrification degree, which reduces the brick’s susceptibility to water absorption as well as weathering and moisture damage^31^. Additionally, as the firing temperature increases from 800 °C to 1100 °C, quartz undergoes structural changes and gradually reacts with alumina phases, contributing to the formation of mullite mineral^32^. This transformation enhances the clay matrix by promoting densification and improved mechanical properties.

**Table 2:** Mixing ratios of acid-activated kaolin, water, and clay used for each brick sample, with corresponding acid type and kaolin/clay proportion.

Sample	Acid type	Acid volume	Water volume	Kaolin used	Clay used	Kaolin/clay ratio for each brick
CK_(H2SO4)_	H_2_SO_4_	10 mL	100 mL	5 g	49 g	1 g kaolin/49 g clay
CK_(HCl)_	HCl	10 mL	100 mL	5 g	49 g	1 g kaolin/49 g clay
CK_(H3PO4)_	H_3_PO_4_	10 mL	100 mL	5 g	49 g	1 g kaolin/49 g clay
CK_(mix)_	H_2_SO_4_ + HCl + H_3_PO_4_	10 mL (total)	100 mL	5 g	49 g	1 g kaolin/49 g clay

### Bricks characterizations

Patterns from 4 to 70˚ 2θ were found using X-ray Diffractometry (XRD) with a Cu target (Model D8, Bruker), which eliminated light on the phase development and mineral structure of the pure and addressed clay compounds. The effects of chemical processing on the structure of clay grains were examined using Fourier transform infrared (FTIR) spectroscopy (Jasco Model 4100, Japan). The IR spectrum produces data with a 4 cm^–1^ recovery in a 4000–400 cm^–1^ region at room temperature (RT). The evaluation of shrinkage behavior resulting from these processes was conducted by analyzing the volumetric shrinkage across all three dimensions of the clay bricks during the drying and firing stages. In order to determine the surface chemical formula of the elements, the mixed bricks’ surface morphology was further investigated using field emission scanning electron microscopy (FESEM, QUANTA 200 FEG from FEI, Japan) fitted with an energy-dispersive X-ray (EDX) spectrometer. Furthermore, the Archimedes method was used to physically measure bulk density and apparent porosity. EN 772-1:2011 was used to evaluate the compressive strength, and each sample’s strength was progressively increased until failure by applying a load centred on its upper surface. After that, each material’s compressive strength was evaluated and given in MPa. A temperature range of 23 to 1000 ˚C was used to conduct a thermal stability examination using the thermogravimetric analyser (TGA) 1600 SETARAM LABSYS Evo. The cooling and heating rates in the air surrounds were both 10 ˚C/min, with an isothermal precision of ± 1 °C. In order to assess the insulation effectiveness, the thermal properties of the burnt clay bricks—such as thermal conductivity, thermal diffusivity, specific heat capacity, and thermal effusivity—were tested using accepted methods. The Hot Disc Thermal Constants Analyser (Hot Disc TPS 2500 S, Göteborg, Sweden) was used to assess the measurement techniques. The transient plane source (TPS) approach, which is widely recognised for its precision and ease of use in examining thermal transport parameters, is utilized by this device. Standard-sized samples were produced, and they were analysed at room temperature. After putting the samples in contact with a sensor, the heat flow response over time was used to compute thermal conductivity. Conversely, one side of the sample was exposed to a heat pulse, and the diffusivity was evaluated by measuring the temperature increase on the other side. In order to estimate the specific heat capacity, the samples were heated and the amount of heat needed to raise their temperature was measured. The density, specific heat capacity, and thermal conductivity of the material were used to compute thermal effusivity.

## Result and discussion

### XRD analysis

**Fig. 3:**
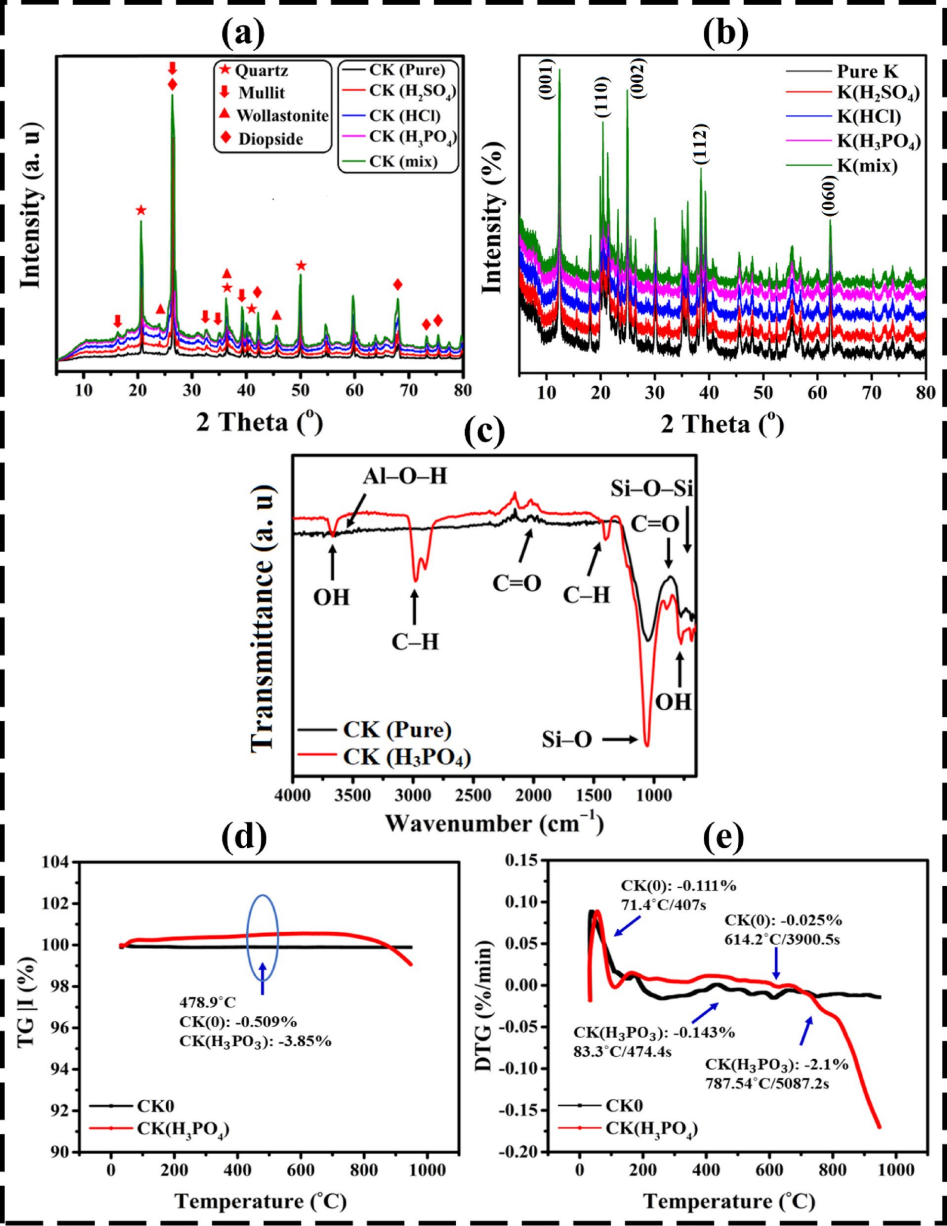
(**a**) XRD patterns of pristine and chemically treated clay brick samples fired at 1100 °C, showing crystalline phase evolution. (**b**) XRD patterns of pure and acid-activated kaolin (H_2_SO_4_, HCl, and H_3_PO_4_) illustrating attenuation of kaolinite crystalline peaks and the emergence of amorphous silica features, confirming structural activation. (**c**) FTIR spectra showing functional group transformations induced by acid activation. (**d**) Thermogravimetric analysis (TGA) and (**e**) derivative thermogravimetry (DTG) curves demonstrating the thermal stability and decomposition behavior of pristine and treated samples.

XRD profiles of the activated clay doped with kaolin and various acids (H_2_SO_4_, HCl, H_3_PO_4_, and their combination) fired bricks were displayed in Fig. 3a. In these bricks that made with and without additives are fired at 1100 °C, the predominant mineral phase is quartz (COD card No: 1011176) 2θ = 21^°^, 28^°^, 36^°^,39^°^, 40^°^, 46^°^, and 50^°^ crystallized in these bricks^33^. Several authors found that an improvement in the mechanical properties through the reduction of the particle size of quartz in fired ceramics^34,35^. Some of the quartz grains particles dissolved and covered with glassy phase at firing temperature of 1100 °C. Increasing the firing temperature therefore increases the glassy phase and vitrification of the fired brick and so reduces the total pore volume, and enhanced the brick strength. Quartz usually present as a crystalline phase in most clays that used in the brick industry^36^. Kaolinite disappears results in the formation of mullite Al_6_Si_2_O_13_ (JCPDS card No: 15–776) 2θ = 17^°^, 26^°^, 33^°^, 37^°^, and 40^°^. Mullite exhibits several important properties, including low thermal conductivity, low thermal expansion, and excellent mechanical performance at elevated temperatures^37^. A high mullite content significantly enhances the refractoriness, refractoriness under load, and resistance to chemical corrosion in refractory bricks. Due to the interlocking nature of mullite crystals within the matrix, mullite-based structures demonstrate superior strength compared to other high-alumina refractories. Mullite refractories are particularly valuable in steelmaking applications, where materials are frequently exposed to slag rich in iron oxides; their strong resistance to iron-oxide-induced corrosion ensures superior durability under such harsh conditions. Additionally, mullite remains structurally stable at temperatures up to 1800 °C^38,39^. The formation of wollastonite, known for its low thermal conductivity, contributes to the improved thermal insulation characteristics of the ceramic matrix^40,41^. Diopside (ICDD 11–654) 2θ = 27^°^, 39^°^, 43^°^, 66^°^, 68^°^, 73^°^, and 75^°^ formed by reaction of quartz with Ca and Mg ions to form CaMg-silicates^41^. Diopside increase products strength^25^, used in the production of insulation ceramics^26,42^, and is important for chemical corrosion resistance characteristics^27^.

### Kaolin activation mechanism and structural evolution

The structural activation of kaolin was verified using XRD and FTIR, both of which clearly illustrate the transformation of kaolinite (Al_2_Si_2_O_5_(OH)_4_) into a less-ordered, more reactive material after acid treatment. The XRD patterns of pure and activated kaolin are shown in Fig. 3b. The pristine kaolin exhibits the typical reflections of highly crystalline kaolinite at 2θ ≈ 12.3^°^, 20.3^°^, 24.9^°^, 38.4^°^, and 62.3^°^, corresponding to the (001), (110), (002), (112), and (060) planes (JCPDS 14–0164). Upon treatment with acids (H_2_SO_4_, HCl, and H_3_PO_4_), these characteristic peaks markedly decreased in intensity and broadened, particularly at 2θ = 12.3^°^ and 24.9^°^, accompanied by the emergence of a diffuse halo centered around 22–25^°^, typical of amorphous silica-rich phases. This transformation indicates the partial dissolution of Al^3+^ from octahedral layers and the collapse of long-range order, signifying the activation of the kaolin lattice^4,5^.

#### Mechanistic interpretation of acid activation

Kaolinite consists of alternating silicate (Si_2_O_5_) and gibbsite-like (Al_2_(OH)_4_) layers held together by hydrogen bonds^43^. The acid activation mechanism proceeds via proton exchange, cation leaching, and framework disintegration, as illustrated schematically below. (i) Proton Exchange and Cation Removal: The acids supply an abundance of H^+^ ions that substitute for exchangeable cations (Na^+^, K^+^, Ca^2+^, Mg^2+^) located on the surface and interlayer positions of kaolinite:

1 $$\equiv Si - O - Mn + + nH^{ + } \to \equiv Si - OH + M_{{(aq)}}^{{n + }}$$

where M^n+^ represents exchangeable cations such as Na^+^, Ca^2+^, or Mg^2+^. This stage increases the surface hydroxyl concentration and surface acidity. (ii) Octahedral Layer Dissolution (De-Alumination): As the attack progresses, Al–O–Si bonds in the octahedral gibbsite layer are hydrolyzed, releasing Al^3+^ ions into the solution. The reaction can be summarized as:

2 $$Al_{2} Si_{2} O_{5} (OH)_{4} + 6H^{ + } \to 2Al_{{aq}}^{{3 + }} + 2SiO_{2} + 5H_{2} O$$

The liberated Al^3+^ leaves behind a porous silica framework with numerous silanol (Si–OH) groups, responsible for the increased surface area and adsorptive capacity of the activated kaolin^8^. (iii) Structural Reorganization and Amorphization: Continued leaching of octahedral Al^3+^ and partial breakdown of the tetrahedral Si–O–Al linkages induce structural reorganization^8^. This generates amorphous aluminosilicate gels and microporous textures, which are confirmed by the disappearance or broadening of the kaolinite XRD peaks and the shift of the baseline between 22–25^°^. The structural transition can be represented as:

3 $$Kaolinite{\text{ }}\left( {crystalline} \right)\left( {H^{ + } /{\text{ }}Acid{\text{ }}attack} \right) \to AmorphousSiO_{2} - Al\left( {OH} \right)_{3} \left( {activated{\text{ }}phase} \right)$$

The formation of these amorphous domains significantly enhances the reactivity and pozzolanic potential of the material^6^. (iv) Specific Role of Each Acid: H_2_SO_4_ acts as a strong oxidizing agent and efficiently removes Al^3+^, forming soluble Al_2_(SO_4_)_3_ complexes.

4 $$2Al^{{3 + }} + 3SO_{4}^{{2 - }} \to Al_{2} (SO_{4} )_{3}$$

HCl promotes selective dissolution of octahedral cations without fully collapsing the tetrahedral sheet, thereby preserving partial order^44^. H_3_PO_4_ not only leaches Al^3+^ but also interacts with the surface to form AlPO_4_-like amorphous species, further weakening the structural integrity and producing the most pronounced amorphization, as evident in XRD by the lowest intensity peaks and a broad hump^45^.

### FTIR assessment

FTIR analyses were conducted on untreated and treated kaolin bricks to examine the chemical properties and structure, bonding, isomorphic replacements, and structural alterations brought about by chemical alterations to clay minerals, as illustrated in Fig. 3c. A variety of bands, ranging from 3695 to 430 cm^–1^, were revealed in the raw brick sample^46^. While the bands at 3420 cm^–1^ and 1620 cm^–1^ correspond to the OH vibrational mode and H–O–H bending of water, respectively, the bands at 3694 cm^–1^ belong to Al–O–H bands^47,48^. In Kaolin, C–H stretching was observed at 2850 cm^− 1^, C–O stretching was assigned to 1095 cm^− 1^, and C = O stretching was recorded at 1821 cm^− 1^^48^. The bands located at 3622 cm^–1^, 1032 cm^–1^, and 920 cm^–1^ represented the dioctahedral smectites. Al–OH bonds were the driving force behind the OH stretching vibration at 920 cm^–1^. The bending vibration of water molecules was measured at 1600–1700 cm^− 1^^49^, that of Si–O bonds at 1040 cm^− 1^ and 690 cm^− 1^, that of Si–O–bending vibration at 795 cm^− 1^^50^, and that of C–H stretching at 2881 cm^− 1^^51^. Quartz was discovered to be present in the bands at 1038, 798, 754, and 690 cm^− 1^, whereas illite and musscovite were found to be at 3620, 914, 795, and 471 cm^− 1^^48^. Feldspar was observed at 1090, 1034, 1011, 790, and 533 cm^− 1^. The band at 1636 cm^− 1^ was a result of physically adsorbed water molecules deforming. Owing to its broad width, this band is probably going to have overlapping bands at 1680 and 1650 cm^− 1^, where water deformation patterns correspond to water in the cation hydration sphere and interlayer cations, respectively^52^. In that order, the Al–O–Si, Si–O–Si, and Si–O bands can be seen at 540, 470, and 430 cm^− 1^^53^.

Following modification in an acid medium containing H_3_PO_4_ solution, some of the bands vanished, were repositioned, or changed in intensity due to acid activation; this suggests a slight disturbance to the crystal structure of the clay. In the first coordination sphere of the interlayer space, there were loosely linked water molecules at 1385 cm^–1^, phosphate ions correlations^54^ and a C = O stretching mode^6^ provided at 937 and 1160 cm^–1^. By activating the solid surface with H_3_PO_4_ acid, H^+^ is adsorbed on the sites, forming an active center surface where the fundamental dissolving process occurs. The group of phosphates was shown to contribute significantly in the 1600–3600 cm^–1^ range because of the P–O–H stretching modes in phosphate that contains hydroxyl^55^. Activation with acid led to the formation of additional bands of Si–O^6^, O–H stretching band^56^, and H–O–H adsorbed water^57^ at 1647, 3380, and 3418 cm^–1^, respectively, as the Si/Al ratio rose. These fluctuations corresponded to changes in shape (i.e., glass formation at very high temperatures) and location of unprocessed kaolinite, which eventually reduces owing to a structural shift in the tetrahedral cations. The leaching process and band shift resulted in the bending vibration mode of physisorbed water on the surface of free silica^6^. In addition, different types of structural OH stretching groups at 2500 and 3425 cm^–1^ were generated by the acid attack and are attributed to the O–H water physisorbed on the surface of the kaolin substance shifting or disappearing^58^. Clay’s hydroxyl resonance bands suggest that protons have attacked the structural OH groups within the mineral layers, causing dehydroxylation, which frees the central atoms from their octahedra, the leaching of Al from the octahedral layers, and a shift of 2881–2499 cm^–1^^59^. The subsequent chemical process is produced when phosphoric acid interacts with the surfaces of Fe and Al minerals, attacking the kaolinite edge^57^.

5 $$\:SOH\: + \:H_{3} PO_{4} \to \:SH_{2} PO_{4} \: + H_{2} O$$

where SOH represents kaolinite’s surface functional group^13^.

Meanwhile, acid treatment contributes to the disappearance of the Si–O–Si absorption band at 470 cm^–1^ in the tetrahedral sheet, hence changing the structure in the tetrahedral cations^45^. A band that developed at 795 cm^–1^ was caused by Al–Mg–OH; however, this band was eliminated as a result of kaolin’s structural shift from crystalline to amorphous phase and reduced Al and Mg levels as a result of acid attack^8^. Moreover, the water physisorbed on the kaolin surface vanishes when additional bands at 1821 and 2925 cm^–1^ are removed. These bands are attributed to the stretching vibrations structural OH of kaolinite as well as acid attack^11,60^. Furthermore, interaction with acids reduced the strength of Al–OH bands at 3669 cm^–1^, displaying deformation of tetrahedral and octahedral layers. H_3_PO_4_ consists of one P = O bond, three P–O bonds, and three O–H bonds^61,62^. The (–OH) groups can create chemical bonds to shape the compound groups, increasing adhesion^63^. Overall, the peaks observed at modified brick in an acid solution of H_3_PO_4_ were identified as variations in crystal structures of kaolinite elements containing phosphate enclosed by water molecules.

Additionally, the FTIR spectra corroborate these structural modifications. The disappearance of the Si–O–Si stretching band at 470 cm^–1^, the reduction of Al–Mg–OH bending at 795 cm^–1^, and the attenuation of Al–OH stretching at 3669 cm^–1^ collectively indicate loss of structural hydroxyls and distortion of the tetrahedral–octahedral framework. The weakening of the –OH bands also confirm the dehydroxylation associated with amorphization. Thus, the combination of XRD, FTIR, and chemical mechanism analysis unequivocally demonstrates that the acid-treated kaolin is structurally activated through de-alumination, surface protonation, and partial amorphization, resulting in a material of higher surface area, increased pore volume, and enhanced reactivity — all essential for improving the performance of the acid-doped clay brick composites.

### Thermogravimetric analysis (TGA)

The thermal stability of the modified and pristine clay combinations in relation to the acids type employed as an activation element was investigated via TGA and DTG approaches, depicted in Fig. 3d and e. As shown in Fig. 3d and e, three major weight-loss regions were observed: (i) below 200 °C, (ii) 200–400 °C, and (iii) 400–900 °C. In the first stage (< 200 °C), the mass reduction corresponds to the loss of physically adsorbed and interlayer water molecules, indicating dehydration of the clay matrix^64^. Between 200 °C and 400 °C, bound hydroxyl groups interacting with interfacial cations undergo dehydration, leading to a reduction in physical weight for the CK(H_3_PO_4_) and CK0 samples of − 3.85% and − 0.509%, respectively. The presence of an activated agent inside the clay matrix caused the shift, and as a result, further functional groups (such as C = O, Si–O–Si, Al–Mg–OH, P = O, P–O, and O–H bonds) were formed. In–situ XRD data substantially support this recent finding^6,65^. With regard to weight loss and decomposition temperature, there is a great deal of resemblance between the TGA curves. This indicates that chain scission and crosslinking in the chemical structure of clay were not significantly impacted by activated phosphoric acid^66^.

Nevertheless, at temperatures between 400 and 750 ˚C, becomes the dominant process, corresponding to the transformation of Al_2_Si_2_O_5_(OH)_4_ → Al_2_Si_2_O_7_ + 2H_2_O^67^. The DTG peaks were found to be tiny and feeble in the pure material, decomposing by − 0.111% at 71.4˚C/407s and − 0.025% at 614.2˚C/3900.5s. In the CK(H_3_PO_4_) scenario, a noticeable reduction in size by − 0.143% at 83.3˚C/474.4s and − 2.1% at 787.54˚C/5087.2s is ascribed to illite dehydroxylation and calcite decomposition^68^. Potential further clay minerals that dehydroxylate in this temperature range consist of smectite and illite/mica, these might very slightly affect the weight loss. The reason behind the rise lies in the high level of acidic medium containing H_3_PO_4_ solution has vanished (i.e., the O–H water physisorbed on the surface of the kaolin material has shifted or disappeared^58^. Meanwhile, phosphoric acid treatment introduced phosphate groups (P–O–Al, P–O–Si) that delayed dehydroxylation, suggesting enhanced structural stability and improved thermal endurance^55^. Conversely, H_2_SO_4_ and HCl treatments facilitated Al^3+^ and Fe^3+^ leaching, promoting amorphous silica formation and increasing microstructural disorder, as evidenced by the intensified DTG peaks around 600–700 °C^69,70^. Beyond 750 °C, decomposition of carbonates and crystallization of high-temperature phases such as mullite and spinel occur. These transformations densify the structure and strengthen interparticle bonding, improving the brick’s ability to resist thermal shock and heat flow. These variations linked to changes in shape (i.e., glass formation at extremely high temperatures) and position of raw kaolinite, which gradually decreases due to a structural shift in the tetrahedral cations. Further, when the Si/Al ratio increased, new bands of Si–O^6^, O–H^56^, and H–O–H adsorbed water^57^ emerged. The leaching process and band shift produced a bending vibration mode of physisorbed water on the surface of free silica^6^. With the CK(H_3_PO_4_) combination, a ramp rate of 10 ˚C/min produced findings similar to nucleation acceleration of mullite or γ-Alumina. Both pure and modified clay solids had residual masses at 25 and 800˚C, thus proves the weight loss in acid-activated clay bricks is rather. Therefore, the stepwise dehydration–dehydroxylation and mineral decomposition processes not only govern mass loss but also dictate the formation of thermally stable phases. The resulting metakaolin and mullite frameworks enhance thermal insulation, heat retention, and long-term stability, confirming that acid activation contributes directly to superior thermal performance and energy efficiency in the final brick structure^68^.

### Shrinkage behavior and its correlation with density and mechanical strength

**Fig. 4:**
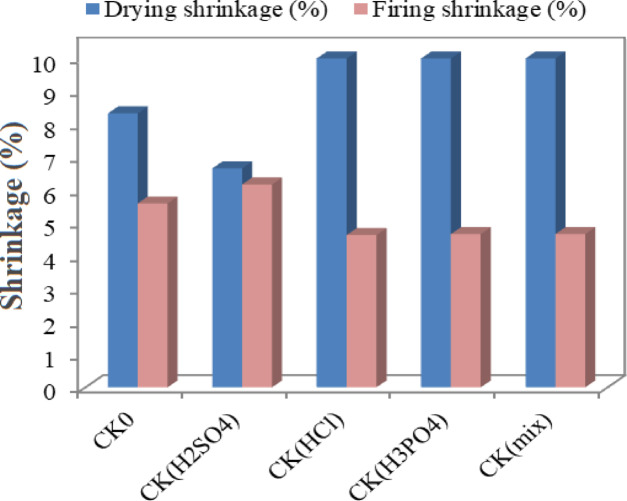
Variation of drying and firing shrinkage (%) of the prepared brick samples as a function of acid type used in kaolin activation (H_2_SO_4_, HCl, H_3_PO_4_, and mixed acids).

The shrinkage behaviour of the bricks is seen in Fig. 4, which varies based on the type of acid used during the firing and drying processes. The behaviour of dried clay objects is significantly influenced by their microstructure during the drying process. Specifically, pore size distribution was found to have an influence on drying shrinkage, whereas porosity was found to have an influence on dry bending strength. Regardless, significant shrinkage is required for the drying behaviour to be effective. The shrinkage in this study grew from 8.33% of the pure compound to 10% with the addition of HCl, H_3_PO_4_, and their combination. The slightly increased drying shrinkage of acid-treated samples is attributed to the enhanced micro-porosity and water retention capacity induced by acid activation, which allows more uniform evaporation and prevents crack formation. Acid leaching dissolved exchangeable cations (Al^3+^, Fe^3+^, and Mg^2+^) and disrupted the kaolinite lattice, leading to finer pore networks and improved structural flexibility during water loss. The way clay shrinks when dried depends on the kind of minerals it contains as well as the quantity of extremely fine particles. Compared to plastic clays, which often crack and distort, sand clays shrink less during drying, drying to a weak and porous body. The removal of water over the drying process causes a ceramic body to shrink; therefore, the amount of water in the material determines how much shrinkage occurs after drying. Throughout the drying process, it’s critical to control the rate of water elimination to avoid interior defects. Whenever drying at temperatures below 50 °C, it is helpful to extend the drying time to increase strength^71^. If a brick’s drying shrinkage is less than 10%, it is usually considered to be of acceptable quality^72^. The drying shrinkage in the samples is appropriate. Both drying and firing shrinkage tests were performed, and all values fell within the acceptable limit of < 10%, as recommended by Zhang, L^73^., confirming the dimensional stability of the fabricated bricks.

At the firing stage, the shrinkage achieved was 5.59% of the pristine combination, which reduced to 4.63% and 4.66%, respectively, with the addition of HCl or H_3_PO_4_, and their combination. This reduction in firing shrinkage corresponds to the enhanced densification and solid-state diffusion facilitated by acid activation, where Fe_2_O_3_ and P-based compounds acted as fluxing and bonding agents, promoting stronger sintering. The formation of mullite phase enhanced the brick’s internal consolidation and mechanical integrity. Consequently, samples with moderate shrinkage exhibited higher apparent density and compressive strength, revealing a direct relationship between shrinkage control, densification, and mechanical performance. The amount of shrinkage that a body experiences after firing is determined by a number of factors, including the volatile contents of the material, the kinds of crystalline phases that make up the material, the degree of dehydration of the clay minerals, and the properties of viscosity and surface tension. It is also possible to express firing shrinkage in terms of linear dimensions, according to Murray^74^. Furthermore, clay-mineral particle size, shape, and chemical alterations all affect shrinkage^71^. Along with contributing to the conversion of additives into ashes, the migration of gases resulting from the breakdown of carbonates and sulphates is the cause of the higher values. As a result of chemical reactions, particle rearrangement, and orientation within the crystal lattice, the solid texture became more compacted during firing, causing shrinkage caused by the removal of residual and chemically combined water and the conversion of additives into ashes^75^. The firing shrinkage of all the moulds was satisfactory, falling below the 8% threshold that is considered the benchmark for high-quality bricks^76^.

### Morphology

**Fig. 5:**
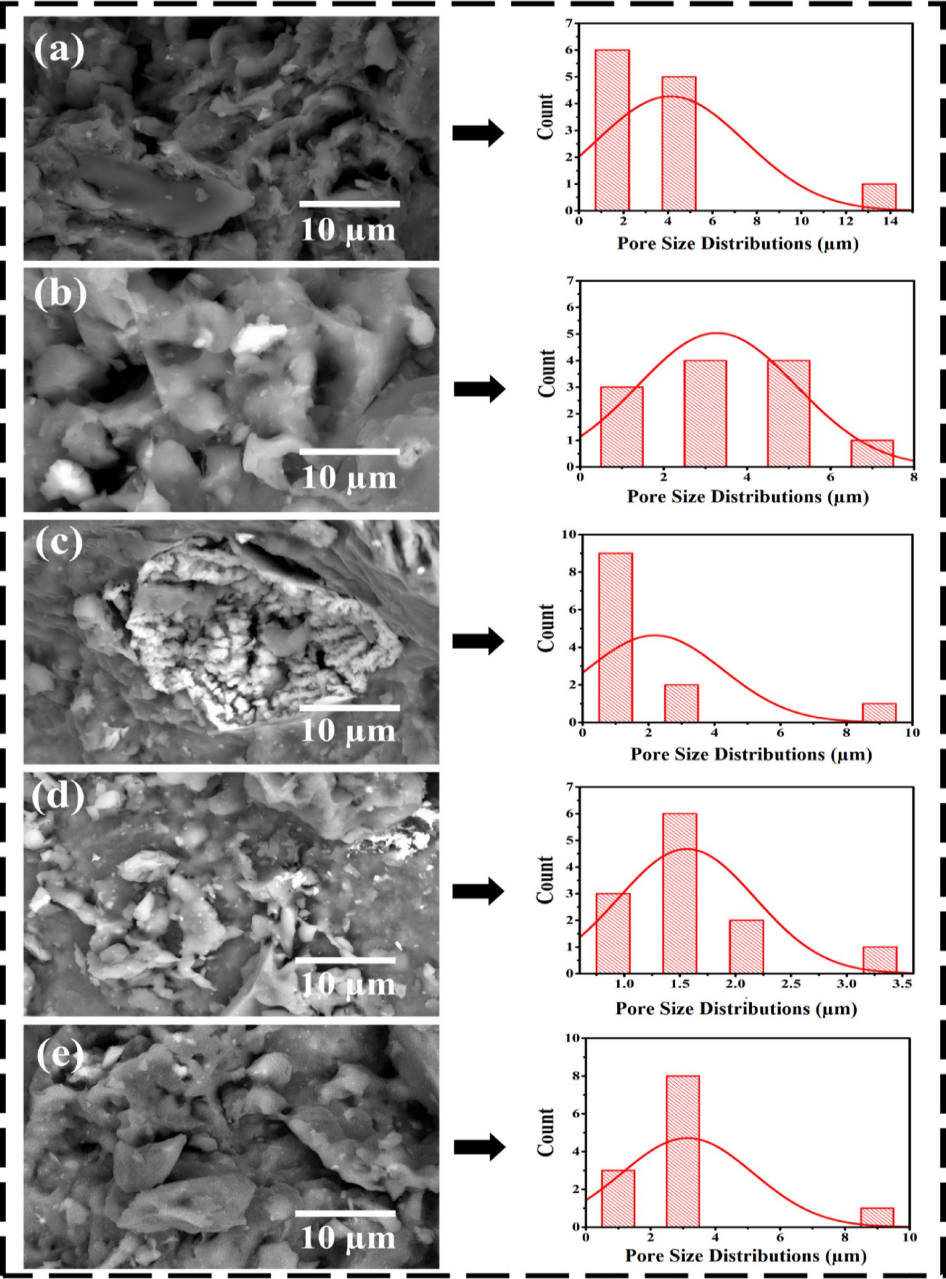
(**a**–**e**) SEM micrographs and corresponding statistical pore size distribution histograms of untreated and acid-treated nanocomposite clays (H_2_SO_4_, HCl, H_3_PO_4_, and mixed acids), showing surface morphology and pore structure modification after activation.

Figure 5a–e show SEM micrographs and pore size distributions of natural and treated clay matrixes with various acid additions. The porosity topography of clay compositions may be noticed based on the kind of acid. More specific, Fig. 5a shows the surface qualities of natural clay with fine pores. Additionally, Fig. 5b–e shows that clay that was treated with acid and applied in a different way has comparable structures. After being acid treated, it became clear that the clay’s structure had strong clay element adhesion due to the copious basic hydroxyl, hydrogen bonds, and other oxygen-related groups that were formed^77,78^.

SEM images revealed that each acid type induced distinctive microstructural modifications within the kaolin–clay matrix. Treatment with H_2_SO_4_ caused partial dissolution of the aluminosilicate lattice, leading to smaller, well-distributed pores and a roughened surface texture. HCl treatment promoted dealumination and silica enrichment, resulting in a dense but highly wrinkled microstructure with increased surface reactivity. In contrast, H_3_PO_4_ formed Al–O–P linkages that reduced pore coalescence and produced the smallest average pore size (≈ 1.41 μm). These transformations confirm that acid attack dismantles the layered kaolinite structure, producing amorphous silica phases and microvoids that enhance both surface area and diffusion pathways. Additionally, depending on the kind of acid, the hybrids’ structure expanded to expose an amorphous structure with more micropores, numerous linkages, and greater internal energy^79,80^. In the meantime, a number of crumpled and wrinkly structures were seen in the clay compositions. This characteristic of clays is essential for avoiding unwanted restacking and sheet aggregation^81^. Accordingly, Fig. 5 shows the statistical estimates of the pore size distribution and average pore sizes of the clay-based kaolin composites both before and after acid treatment^82^. When H_2_SO_4_ acids were processed inside the clay structure CK_(H2SO4)_, the estimated average pore size of CK0 was 3.98 $\:\pm\:$ 0.16 μm, and it decreased to 3.02 $\:\pm\:$ 0.12 μm. Other acids (such 2.05 $\:\pm\:$ 0.08 μm at CK_(HCl)_ and 1.38 $\:\pm\:$ 0.06 μm at CK_(H3PO4)_ samples) also showed same declining trend. However, when a mixed-acid system containing H_2_SO_4_, HCl, and H_3_PO_4_ was used, the average pore size increased slightly to 3.01$\:\pm\:$ 0.12 μm. This moderate increase is attributed to the synergistic action of the three acids, where simultaneous dealumination, Fe^3+^ leaching, and phosphate bonding reactions create a heterogeneous dissolution environment. The competing ionic species hinder complete structural collapse, yielding hierarchical micro- and mesopores that connect through intergranular channels. Such multiscale pore formation enhances moisture transport and thermal buffering, thereby improving the bricks’ thermal insulation characteristics. These discoveries might be clarified through the formation of novel intermolecular covalent and hydrogen interactions among clay–H_2_O, Al–Mg–OH, P = O, P–O, and –OH groups^6,83,84^. These linkages control the rate of hydration crystal formation, resulting in a homogeneous distribution of clay elements^85^. The upshot is that these composites’ surface roughness and thermal properties were substantially enhanced as a result of the active ingredients’ miscibility.

### EDX observations

**Fig. 6:**
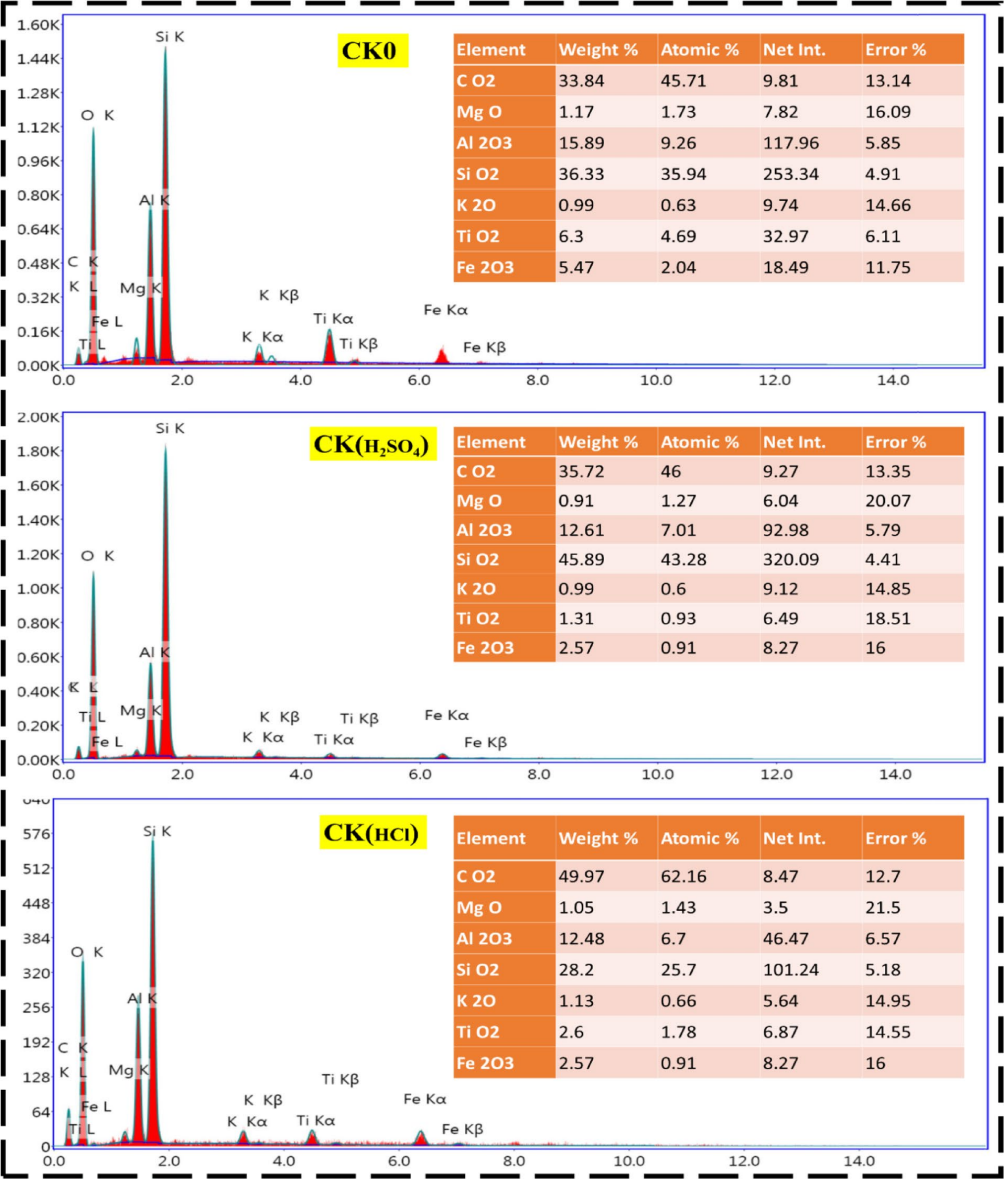

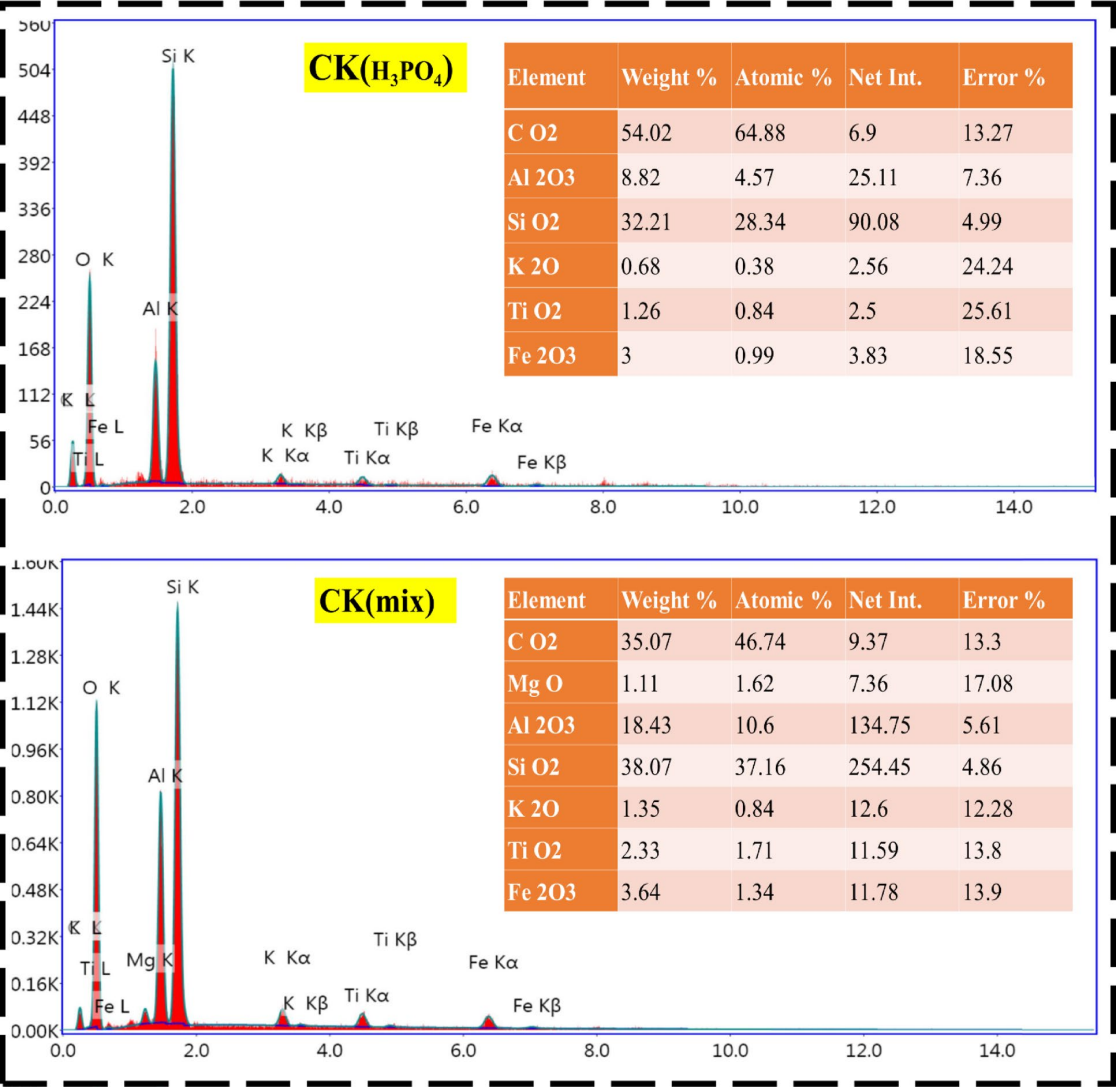
EDX spectra of untreated and acid-treated nanocomposite clays using different acids (H_2_SO_4_, HCl, H_3_PO_4_, and mixed acids), illustrating the elemental composition and chemical changes following activation.

In the context of fired clay-based composite bricks, EDX mapping provides valuable insights into how different chemical treatments—such as doping with kaolin and various acids (H_2_SO_4_, HCl, H_3_PO_4_, and their combination)—influence the thermophysical properties of the bricks. The study focuses on different formulations, including CK0, CK_(H2SO4),_ CK_(HCl),_ CK_(H3PO4),_ and CK_(mix)_ composites (See in Fig. 6). In particular, EDX mapping demonstrated the presence of elements associated with kaolin (e.g., CO_2_, MgO, AL_2_O_3_, SiO_2_, K_2_O, TiO_2_, and Fe_2_O_3_) when kaolin is doped into clay and treated with H_2_SO_4_^86^. These elements possessed varying weights of 35.72, 0.91, 12.61, 45.89, 0.99, 1.31, and 2.57 wt%, respectively, along with a detectable amount of sulfur, confirming the incorporation of sulfate functional groups (Al–O–SO_4_ and Si–O–SO_3_) throughout the matrix. These sulfate linkages act as bridging sites between silica and alumina phases, promoting enhanced crystallization control, lower defect density, and improved mechanical bonding during firing^87^. In the case of clay doped with kaolin and treated with HCl, EDX mapping revealed a chlorine distribution alongside kaolin elemental signatures (CO_2_, MgO, AL_2_O_3_, SiO_2_, K_2_O, TiO_2_, and Fe_2_O_3_)^88^.Chlorine incorporation likely arises from transient formation of metal chlorides (AlCl_3_, FeCl_3_), which decompose during heating but enhance mass transport, favouring sintering and densification. The uniform Cl signal suggests that chloride ions penetrated interlayer regions, assisting in the redistribution of cations and yielding a more homogeneous structure^89^. Following treatment with H_3_PO_4_, EDX mapping showed phosphorus in addition to the kaolin-associated elements^79^. Phosphoric acid treatment resulted in the in-situ formation of aluminum phosphate (AlPO_4_) and silicon phosphate (SiP_2_O_7_) phases. These phosphate linkages significantly enhance the thermal stability and acid resistance of the brick, as they chemically fix phosphorus within the matrix and impede grain growth. A uniform P distribution indicates efficient crosslinking, contributing to improved compressive strength and reduced thermal conductivity^90^. For the mixed-acid composite CK_(mix)_, the EDX mapping revealed the concurrent presence of sulfur, chlorine, and phosphorus signals homogeneously dispersed across the clay matrix. This demonstrates that the acids collectively modified the lattice through overlapping reactions—sulfate ions forming structural crosslinks, chloride ions enhancing ionic mobility, and phosphate ions providing long-term structural fixation. The synergistic incorporation of these acid-derived elements generated a hybrid aluminosilicate–sulfate–phosphate framework with interconnected microvoids, which balance strength with thermal insulation. The even dispersion of S, Cl, and P indicates that these species are chemically bonded rather than merely surface-deposited, leading to durable, chemically stable, and thermally resistant composite bricks.

A uniform distribution of phosphorus, as seen in EDX mapping, would suggest effective chemical modification, potentially leading to enhanced thermal resistance and mechanical properties^6^. The phosphate compounds formed can create stronger bonds within the clay matrix, improving its overall stability and resistance to thermal degradation. Moreover, the microstructural changes induced by phosphoric acid resulted in a denser, more robust material with lower thermal conductivity^66^. On the other side, the most complex formulation involves a combination of H_2_SO_4_/HCl/H_3_PO_4_ acids (CK_(mix)_). EDX mapping for this composite showed the distribution of CO_2_, MgO, AL_2_O_3_, SiO_2_, K_2_O, TiO_2_, and Fe_2_O_3_ within the clay matrix. The combined acid treatment was expected to produce a highly modified clay structure, with each acid contributing to different aspects of the material’s properties. The EDX maps revealed how sulfur, chlorine, and phosphorus elements were distributed and whether they are uniformly integrated into the clay. A well-distributed presence of all three elements indicated that the combined acid treatment has effectively modified the clay on multiple levels, potentially leading to significant enhancements in thermophysical properties^83^. The combination of these acids resulted in a complex interplay of chemical reactions, leading to the formation of sulfate, chloride, and phosphate compounds within the clay. This could enhance the bricks’ thermal stability, reduce thermal conductivity, and improve mechanical strength. Therefore, the results suggest that the choice of acid, as well as the combination of acids, plays a critical role in determining the final properties of the composite bricks, with the potential for tailored material properties depending on the specific application requirements.

### Porosity and density properties

**Fig. 7:**
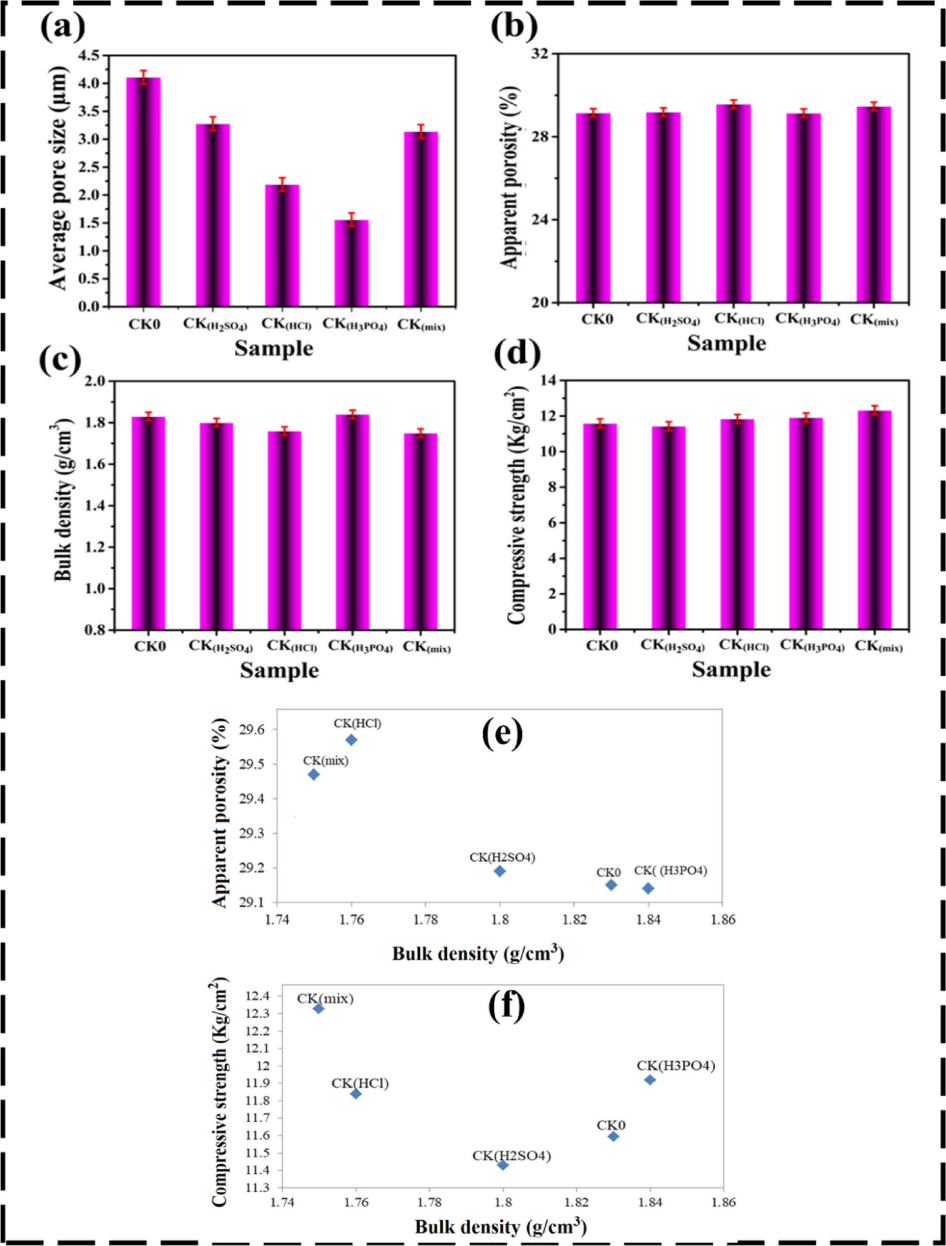
(**a**) Average pore size, (**b**) Apparent porosity, (**c**) Bulk density, (**d**) Compressive strength, (**e**) Correlation between apparent porosity and bulk density, and (**f**) Relationship between bulk density and compressive strength of the clay–acid brick samples, emphasizing the interdependence of structural and mechanical properties.

The kaolinite-based fired clay bricks synthesized in this study exhibited enhanced porosity, density and mechanical properties due to the incorporation of various acid activators, as seen in Fig. 7. Specifically, the production of Clay@Kaolin composites with H_2_SO_4_, HCl, and H_3_PO_4_, including a mixed system of all three acids, resulted in apparent porosity values ​​of 29.15%, 29.19%, 29.57%, 29.14%, and 29.47%, respectively. The slight variations in porosity highlight the sensitive influence of acid treatments on the internal brick structure^91^. At the atomic and microstructural scale, acid activation of kaolin initiates a series of dissolution, ion-exchange, and dealumination processes that substantially modify both the crystal structure and the physicochemical characteristics of the clay minerals. During treatment, H⁺ ions from the acid solutions replace exchangeable cations (Na^+^, K⁺, Ca^2+^, and Mg^2+^) within the interlayer and structural sites of kaolinite, promoting the leaching of Al^3+^ from both octahedral and tetrahedral sheets. This process disrupts the Al–O–Si framework and leads to the partial amorphization of the layered silicate structure^10,92^. The resulting removal of Al–OH and Si–O–Al linkages generate additional micro- and mesoporous sites, thereby enhancing the overall surface area and internal pore volume. The structural response of kaolin varies depending on the type and strength of acid used. HCl, being a strong non-oxidizing acid, primarily attacks octahedral Al^3+^ centers, dissolving up to 90% of octahedral aluminum and yielding a silica-enriched residue characterized by increased microporosity and high specific surface area^18^. Sulfuric acid (H_2_SO_4_), due to its bisulphate anion, enhances dealumination efficiency and induces partial leaching of Fe and Al, which further weakens the aluminosilicate bonds and results in a more disordered amorphous silica network^58,93^. In contrast, H_3_PO_4_ interacts differently by forming Al–O–P linkages through condensation reactions and simultaneously leaching Fe^3+^ impurities from the clay matrix^12^. This dual action not only purifies the structure but also introduces phosphate phases that enhance the reactivity and structural stability of the final fired product.

The combined use of mixed acids intensifies these mechanisms synergistically. The coexistence of Cl^–^, $\:{\mathrm{S}\mathrm{O}}_{4}^{2-}$, and $\:{\mathrm{P}\mathrm{O}}_{4}^{3-}$ ions result in complex dissolution–precipitation equilibria, which create hierarchical pore structures and heterogeneous nucleation centers for new mineral phases during the firing stage. When the treated kaolin is fired at 1100 °C, reorganization of the amorphous silica network leads to the formation of diopside. The newly formed mineral interacts with the remaining kaolinite phases, filling interparticle voids while simultaneously generating microcracks and pores that regulate water transport and mechanical interlocking. Consequently, acid activation plays a dual role: (i) enhancing porosity through structural dissolution and ion leaching, and (ii) improving mechanical cohesion via secondary mineral formation upon firing. The partial amorphization of kaolin increases the number of reactive sites available for silicate polymerization, while the controlled precipitation of diopside and mullite reinforces the brick matrix through solid-state sintering, leading to improved structural cohesion and enhanced mechanical performance. The final microstructure, characterized by interconnected pores and dense bonding regions, supports optimal water retention and thermal regulation within the fired clay body. This balance between porosity and strength is crucial for achieving lightweight, thermally efficient, yet mechanically robust construction materials^94^. The introduction of these activators facilitated the controlled formation of two new mineral phases within the brick matrix—mullite and diopside. These minerals are critical as they create unique interactions with the kaolinite and pure clay components, leading to more controlled water retention within the composite material^95,96^. This improved moisture management supports better thermal activity, enabling more efficient heat transfer and insulation performance^97^. Overall, the addition of kaolin and specific acid activators proved effective in optimizing both microstructural and thermophysical properties of the fired clay bricks, enhancing their potential use in energy-efficient construction^98^.

In a similar vein, the kaolinite-based bricks demonstrated notable trends in bulk density due to the incorporation of different acid activators, as displayed in Fig. 7c. The samples—CK_0_, CK_(H2SO4),_ CK_(HCl),_ CK_(H3PO4),_ and CK_(mix)_—achieved bulk densities of of 1.83 g/cm^3^, 1.80 g/cm^3^, 1.76 g/cm^3^, 1.84 g/cm^3^, and 1.75 g/cm^3^, respectively. These variations reflect the role of each acid in influencing the compaction and overall structure of the clay matrix. The highest bulk density was observed for CK_(H3PO4)_ (1.84 g/cm^3^), indicating dense sintering, whereas the lowest bulk density (1.75 g/cm^3^) in CK(mix) suggests a more porous microstructure possibly due to the formation of glassy phases^89,99–101^. Although the variation in bulk density is only about 4.5%, it is consistent with prior studies, where significant shifts in density are typically observed above 1100 °C, due to glassy-phase transitions^102^. These outcomes demonstrate that acid activators can be strategically selected to tailor brick density—and by extension, their thermal and mechanical behavior—for specific performance demands in sustainable architecture.

### Mechanical properties

Regarding mechanical performance, the modified bricks exhibited a progressive and consistent enhancement in compressive strength following acid activation (Fig. 7d). The measured values for CK0, CK_(H2SO4)_, CK(HCl), CK_(H3PO4)_, and CK_(mix)_ were 11.59, 11.43, 11.84, 11.92, and 12.33 kg/cm^2^, respectively. Although the overall increment of approximately 6% from CK0 to CK(mix) may appear modest, it signifies a meaningful improvement in the microstructural integrity and load-bearing capacity of the bricks, particularly given the low-cost raw materials and moderate firing temperature used in fabrication^5,103^. The observed strengthening can be directly correlated to the formation of thermodynamically stable mineral phases—primarily diopside (CaMgSi_2_O_6_), and mullite (3Al_2_O_3_·2SiO_2_)—that emerged as a consequence of acid-induced activation and subsequent thermal reactions. Acid activation promotes selective leaching of alumina and silica species, increasing the availability of reactive oxides for silicate crystallization during firing. Among the acids used, H_2_SO_4_ was most effective in dissolving Al^3+^ and Fe^3+^ oxides, whereas HCl promoted cation exchange with Ca^2+^ and Mg^2+^ species, enhancing diopside nucleation. In contrast, H_3_PO_4_ introduced phosphate species that acted as fluxing agents, lowering the vitrification temperature and facilitating phosphate–silicate bonding^56^. When these acids were combined, their synergistic leaching, fluxing, and catalytic effects yielded optimal conditions for nucleation and crystal growth, explaining the superior strength and densification observed in CK_(mix)_.

The diopside phase plays a critical role in improving both mechanical and thermophysical properties. As a magnesium-calcium silicate mineral with a relatively low melting point among CaO–MgO–Al_2_O_3_–SiO_2_ systems, diopside enables enhanced densification at lower sintering temperatures, leading to reduced porosity and higher matrix cohesion^104,105^. Its fine-grained crystalline structure acts as a skeleton reinforcing the clay matrix, while its intrinsic thermal stability and low dielectric loss reduce microcrack formation during thermal cycling. These properties make diopside an effective strength enhancer and thermal stabilizer, particularly relevant for building applications in hot climates^106^. Mullite has great relevance in both traditional and advanced ceramics. This is mostly due to its features, which include minimal thermal expansion, strong creep resistance, thermal stability, conductivity, and corrosion resistance. Its fracture toughness and mechanical strength are also satisfactory. Mullite is a crystalline phase formed by heating clay materials. Bending strength improves with mullite %. As the mullite percentage increases, so does the microstructure, which becomes finer^107^.

The acid activators also promote the formation of a transient glassy phase during sintering, which fills voids and reinforces the particle network. This glassy intergranular phase, in conjunction with the crystalline diopside and mullite, facilitates a densification mechanism governed by viscous flow and solid-state diffusion, resulting in enhanced hardness and dimensional stability. Among the acids, phosphoric acid (H_3_PO_4_) was particularly effective in this regard, as it generated phosphate-based amorphous phases that contributed to surface vitrification and improved the bonding strength at the grain interfaces^108,109^. Furthermore, the conversion of kaolinite into mullite and secondary aluminosilicate phases plays a central role in the overall mechanical and thermal performance. Al_2_O_3_, SiO_2_, and Fe_2_O_3_, the principal oxides in kaolin, undergo partial rearrangement or leaching during acid activation. This modification alters the Si/Al ratio, facilitating mullite crystallization and the stabilization of the matrix against thermal shock. The presence of Fe_2_O_3_ further aids sintering by forming solid solutions with mullite, leading to a tighter microstructure and improved compressive strength, particularly at calcination temperatures near 1100 °C.

Ultimately, the synergistic interaction of acid activators, the formation of strengthening crystalline phases (diopside, and mullite), and the development of a continuous glassy network collectively enhance both mechanical robustness and thermal insulation efficiency. These results confirm that acid activation not only optimizes the phase transformation and densification behavior of fired clay but also promotes the design of thermally efficient, sustainable building materials with extended durability and reduced energy requirements for cooling and heating applications^110^.

To further understand the structural behavior of the fabricated bricks, the relationships between apparent porosity and bulk density, as well as bulk density and compressive strength, were graphically presented in Fig. 7e and f, respectively. These plots align with trends reported in literature^111,112^, confirming that increased porosity leads to reduced density, which in turn influences the mechanical strength of the bricks. The development of compressive strength in acid-activated clay bricks, despite modest numerical gains, can be justified by microstructural and compositional transformations. Acid treatment alters the kaolinite structure through ion exchange and leaching of Al^3+^, which leads to increased porosity and reduced bulk density, forming a lightweight, thermally insulating matrix^10,11^. Although higher porosity generally reduces strength, the formation of secondary mineral phases like mullite during sintering improves mechanical integrity by enhancing crystal bonding and densification^104,105^. The incorporation of Fe_2_O_3_ facilitates sintering consolidation, further supporting compressive strength^108^. Graphical analysis (Fig. 7e and f) highlights clear inverse and direct relationships between porosity-density and density-strength, respectively, consistent with literature findings^57,113^. Thus, the strength improvement is attributed to a balance between reduced density from acid leaching and structural gains from enhanced mineral phase development^114^. Overall, although moderate in variation, confirm that acid activators play a foundational role in enhancing the mechanical strength and microstructural quality of kaolinite-based bricks. Even small improvements at this stage are vital in a stepwise material optimization process, supporting broader efforts to develop durable, sustainable, and energy-efficient construction materials^57^.

### Thermophysical properties of designed clay bricks

**Fig. 8:**
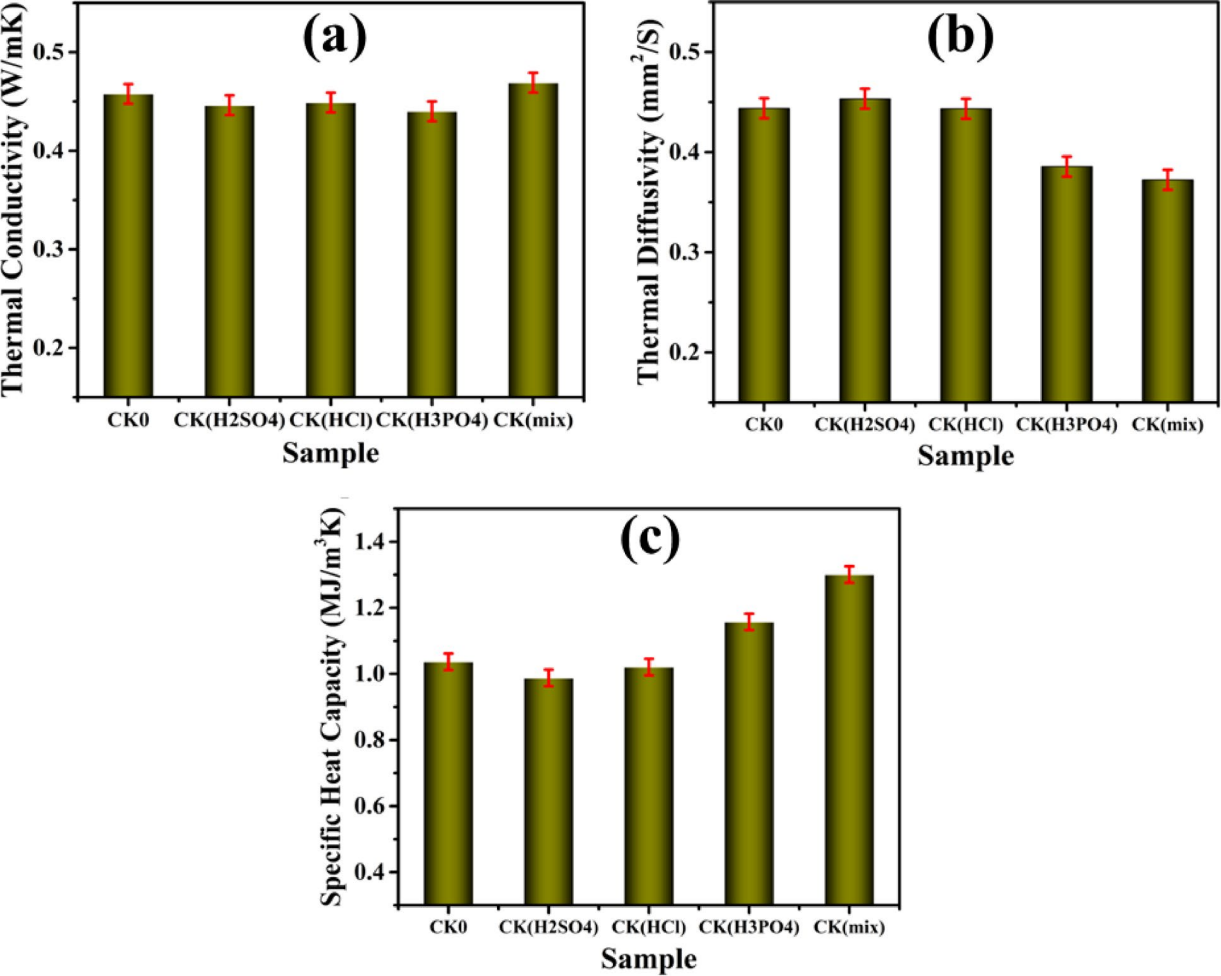
Thermophysical properties of the clay–acid composite brick samples: (**a**) Thermal conductivity, (**b**) Thermal diffusivity, and (**c**) Specific heat capacity, demonstrating the influence of acid activation on heat transfer performance.

The reduction of thermal conductivity, diffusivity, and specific heat capacity in fired clay-based composite bricks is essential for improving their insulating capabilities. These thermophysical properties were enhanced through different doping levels, including CK0, CK_(H2SO4),_ CK_(HCl),_ CK_(H3PO4),_ and CK_(mix)_ composites, as shown in Fig. 8. Thermal conductivity reduced following the various treatments with H_2_SO_4_ and HCl; the smallest value was 0.44 W/mK generated via the process with H_3_PO_4_ acid. Whenever kaolin-modified phosphoric-based materials are produced and elevated to high temperatures, water that is chemically bound in the hydrated substance grows free and migrates into the pores^57^. This migration is caused by the lower permeability of brick-like clay substances, which affects the capacity of water to move out via their pores, resulting in raised pore pressure, which increases as temperature rises. The increase in vapour pressure continues until the thermal stress is sufficient to cause explosive spalling of the fired bricks. This could result in the lowest thermal conductivity among the composites, making it an excellent insulator. Benk and Coban’s observations are supported by the explosive spalling’s rise in accessible microporosity and subsequent reduction in thermal conductivity^115^. Likewise, the rate of transferring heat within a substance that referred to thermal diffusivity was reduced via the chemical treatments with HCl and H_3_PO_4_. As a good insulator, the mix acids reached the lowest value of 0.3724 mm^2^/S, due to the multiple acid treatments, which create a complex microstructure that significantly impedes heat flow. On the other hand, the total amount of energy maintained per unit weight, or specific heat capacity, is dictated by the form and composition of the brick substance. The durability of the brick is largely dependent on how well the clay brick materials retain heat. The highest specific heat capacity was found in tests treated with mix acids^116^. High specific heat capacity values result in small indoor temperature variations, decrease heat transfer stages, and have a beneficial effect on thermal comfort as they reduce interior fluctuations in temperature. Specific heat capacity increases when acid treatment raises SiO_2_ level, as reported by Mako et al.^117^. In light of the air’s relatively low thermal conductivity (0.025 W/mK), the penetration of acids raised the porosity overall, which results in a higher number of pores along with low thermal conduction^118^. Further, the air has an additional specific heat than clay, so among the most significant elements influencing it is the brick’s microstructure^119^. The process of acid activation modifies clays by increasing their surface area, pore volume, and pore diameters. It also dissolves structural ions and rearranges the clay’s structure. Moreover, the acid treatment substitutes H^+^ ions for the various exchangeable cations and gradually releases Al^3+^ from both tetrahedral and octahedral sites^10^.

Indeed, porosity is a critical parameter influencing the overall heat transport behavior of fired clay-based composite bricks. High apparent porosity facilitates the entrapment of air—an excellent thermal insulator (thermal conductivity ≈ 0.025 W/mK)—within the pore network, thereby reducing solid–solid contact and lowering the effective thermal conductivity of the brick^57^. The uniform distribution of these pores is equally essential, as it minimizes continuous heat conduction pathways and enhances the homogeneity of insulation performance. Following acid activation, significant structural and compositional changes occur within the kaolinite matrix. The HCl treatment, in particular, yielded the highest apparent porosity, which is attributed to the dissolution and leaching of Al^3+^ ions from both tetrahedral and octahedral sites. This ion removal disrupts the aluminosilicate framework, leading to increased pore formation and visible changes in color, density, and microstructure. Consequently, the material transitions into a more porous and lightweight solid, which effectively suppresses heat conduction^11^. Furthermore, the mixed-acid system (H_2_SO_4_/HCl/H_3_PO_4_) achieved the lowest bulk density, primarily due to the extensive elimination of metallic oxides and the creation of a more open, interconnected pore structure. This decrease in density corresponds directly to the enhanced thermal insulation capacity of the bricks, as denser materials typically facilitate faster heat transfer. The acids also induce partial dehydroxylation and structural rearrangement, promoting microstructural refinement and controlled pore evolution that collectively reduce thermal diffusivity. In addition, the specific heat capacity of the acid-modified composites exhibited a modest increase, especially in the mixed-acid sample, which can be linked to the higher SiO_2_ content and trapped air volume within the microstructure. A higher specific heat capacity indicates the material’s ability to absorb more energy before its temperature rises, thus improving thermal comfort and stability under fluctuating environmental conditions. Therefore, acid activation—particularly using a combined mixture of H_2_SO_4_, HCl, and H_3_PO_4_—optimizes the pore structure, decreases bulk density, and enhances the overall insulating efficiency of fired clay bricks. The synergistic modification of porosity, diffusivity, and heat capacity through controlled mineral leaching and microstructural tailoring yields an advanced thermally efficient composite material ideal for sustainable building applications^120,121^.

### Sustainability implications of chemically activated bricks in hot climates

Chemically activated bricks exhibit significant advantages in sustainable construction, particularly for hot and arid environments such as Upper Egypt. Their superior thermal insulation properties reduce the cooling energy required in buildings by minimizing heat transfer through walls. The dense microstructure and optimized porosity achieved through acid activation (H_2_SO_4_, HCl, or H_3_PO_4_, or mixed systems) effectively lower the thermal conductivity and diffusivity, contributing to improved indoor comfort without reliance on mechanical cooling systems. The environmental advantages are further amplified by reduced carbon emissions during manufacturing. Unlike conventional fired bricks, which require prolonged kiln operations exceeding 1000 °C, chemically activated and partially stabilized systems (e.g., CSEBs) can be produced at much lower temperatures or without firing, thereby cutting CO_2_ emissions by 60–80%^122,123^.

Sustainability assessment criteria that can be applied to evaluate their environmental impact include:

- Embodied energy and CO_2_ emissions: Quantifying the total energy used and greenhouse gases emitted during production.
- Thermal performance indicators: Such as thermal resistance, conductivity, and effusivity, which determine energy-saving potential in buildings.
- Water and energy use efficiency: Measuring resource intensity during processing and curing.
- Raw material circularity: Incorporation of recycled plastics, agricultural residues, or local soils reduces environmental burden and supports waste valorization.
- Lifecycle durability and recyclability: Ensuring long-term performance and the ability to reuse or recycle materials at end-of-life stages.

In the climatic context of Upper Egypt, where high solar radiation and extreme diurnal temperature variations prevail, these features offer considerable reductions in operational energy demand and CO_2_ emissions^124^. Moreover, using locally sourced and waste-based materials reduces transportation costs, promotes regional economic development, and aligns with the UN Sustainable Development Goals (SDGs 7, 11, and 13) related to affordable clean energy, sustainable cities, and climate action^125,126^. Therefore, chemically activated bricks serve not only as thermally efficient materials but also as a cornerstone for eco-friendly, resource-conserving, and climate-adaptive construction strategies in hot regions^127^.

## Conclusion

This study clearly demonstrates that doping fired clay-based composite bricks with kaolin modified using different acid treatments—namely H_2_SO_4_, HCl, H_3_PO_4_, and their mixture—leads to significant improvements in thermophysical properties, making these materials highly suitable for thermal insulation applications. The most pronounced enhancement was observed in thermal conductivity, which decreased from 0.46 W/mK in the untreated brick to 0.44 W/mK in the sample treated with H_3_PO_4_. Furthermore, the composite treated with the mixture of all three acids achieved the lowest thermal diffusivity, measured at 0.3724 mm^2^/s, suggesting a strong resistance to heat transfer and improved insulating behavior. These improvements are largely attributed to structural modifications induced by acid activation, which increase the porosity and disrupt heat flow pathways. In terms of specific heat capacity, the samples treated with the mixed acids displayed the highest values, indicating an enhanced ability to store thermal energy and thereby stabilize indoor temperatures under variable climatic conditions. This enhancement is closely linked to increased SiO_2_ content and the development of a more porous matrix, as confirmed by chemical analysis and microstructural observations.

The acid treatments also led to slight but significant increases in apparent porosity, ranging between 29.15% and 29.47%, without causing structural collapse. Interestingly, despite this increase in porosity, the compressive strength of the bricks remained within a practical range (11.59–12.33 kg/cm^2^), which confirms the mechanical reliability of the modified materials. XRD analysis revealed the formation of two key mineral phases—mullite and diopside—within the acid-activated matrix. The presence of mullite contributed to improved cohesion among the clay particles, promoting strength and structural integrity, while diopside, known for its application in insulation ceramics, enhanced the chemical durability and thermal resistance of the composites. These microstructural and mineralogical evolutions are central to the observed improvements in thermal and mechanical performance. Overall, the combination of H_2_SO_4_, HCl, and H_3_PO_4_ produced a synergistic effect that yielded the best-performing brick in terms of low thermal conductivity, reduced diffusivity, high specific heat capacity, and acceptable compressive strength. These findings underscore the potential of acid-activated kaolin-based clay composites for applications in energy-efficient and sustainable construction. Future work is recommended to explore the long-term durability, environmental compatibility, and industrial scalability of these novel materials to fully validate their practical application in modern building envelopes.

## Data Availability

All data generated or analysed during this study will be available on request.
